# Validation of Modified Objective Prognostic Score in Patients with Advanced Cancer in Taiwan

**DOI:** 10.1089/pmr.2024.0036

**Published:** 2024-09-30

**Authors:** Yusuke Hiratsuka, Sang-Yeon Suh, Seok Joon Yoon, Shao-Yi Cheng, Sung-Eun Choi, Sun Hyun Kim, David Hui, Ping-Jen Chen, Hsien-Liang Huang, Jen-Kuei Peng, Masanori Mori, Takashi Yamaguchi, Isseki Maeda, Satoru Tsuneto, Tatsuya Morita

**Affiliations:** ^1^ Department of Palliative Medicine, Takeda General Hospital, Aizuwakamatsu, Japan.; ^2^ Department of Palliative Medicine, Tohoku University Graduate School of Medicine, Sendai, Japan.; ^3^ Department of Family Medicine, Dongguk University Ilsan Hospital, Seoul, South Korea.; ^4^ Department of Medicine, Dongguk University Medical School, Seoul, South Korea.; ^5^ Department of Family Medicine, Chungnam National University Hospital, Daejeon, South Korea.; ^6^ Department of Family Medicine, College of Medicine and Hospital, National Taiwan University, Taipei, Taiwan.; ^7^ Department of Statistics, Dongguk University, Seoul, South Korea.; ^8^ Department of Family Medicine, School of Medicine, Catholic Kwandong University International St. Mary’s Hospital, Incheon, South Korea.; ^9^ Department of Palliative Care, Rehabilitation and Integrative Medicine, The University of Texas MD Anderson Cancer Center, Houston, Texas, USA.; ^10^ Department of Family Medicine, Kaohsiung Medical University Hospital, and School of Medicine, Kaohsiung Medical University, Kaohsiung, Taiwan.; ^11^ Division of Palliative and Supportive Care, Seirei Mikatahara General Hospital, Hamamatsu, Japan.; ^12^ Department of Palliative Medicine, Kobe University Graduate School of Medicine School of Medicine, Kobe, Japan.; ^13^ Department of Palliative Care, Senri-Chuo Hospital, Toyonaka, Japan.; ^14^ Department of Human Health Sciences, Graduate School of Medicine, Kyoto University, Kyoto, Japan.

**Keywords:** advanced cancer, palliative care, prognostication, validity

## Abstract

**Background:**

Modified versions of the Objective Prognostic Score (mOPS) needs to be validated to reflect practical palliative care circumstances in Taiwan.

**Objectives:**

We compared the abilities of an mOPS score of 1.5 or higher versus a Karnofsky Performance Status (KPS) score of 30 or lower to predict 2-week mortality in patients with advanced cancer in Taiwan.

**Design:**

Observational study.

**Setting/Subjects:**

We performed a secondary analysis of an international multicenter cohort study of patients in East Asia. Participants were inpatients with advanced cancer in palliative care units (PCUs) in Taiwan.

**Measurements:**

We compared the mOPS-B model, which does not require laboratory tests, with the KPS in a 2-week survival timeframe. We compared the accuracy of the prognostic models using sensitivity, specificity, and area under the receiver operating characteristic curve (AUROC). Calibration plots and net reclassification indices (NRI) for 2-week survival were compared between the two models. Differences in survival between the higher- and lower-scoring groups of each model were identified using the log-rank test.

**Results:**

We included 317 patients, with a median survival of 14.0 days. The mOPS-B had a high sensitivity (0.82) and high AUROC value (0.69). By contrast, the KPS demonstrated good sensitivity (0.77) and an acceptable AUROC value (0.65) for predicting 2-week survival. The calibration plot did not demonstrate satisfactory agreement between the actual and predicted survival times in either the mOPS-B or the KPS groups. Our NRI was positive (absolute value: 22%), indicating that mOPS-B predicted 2-week survival better than KPS.

**Conclusions:**

The mOPS-B may serve better than the KPS as a screening tool for admission to PCUs in Taiwan because it was more accurate at predicting 2-week survival.

## Key Message

The modified version of the objective prognostic score without laboratory data (mOPS-B) has a high sensitivity and discrimination ability. We suggest using the mOPS-B as a screening tool for admission to palliative care units in Taiwan.

## Introduction

Prognostic information is essential for patients with advanced cancer, their families, and health care providers. It is fundamental to decision making related to systemic anticancer treatment and palliative care.^1^ Although clinicians’ prediction of survival (CPS) is easy to use, it is optimistic in palliative care.^2^ Thus, many prognostic tools have been developed to complement the inaccuracy of CPS. The Objective Prognostic Score (OPS) was developed in Korea to predict 3-week survival.^3^ It consists of seven items and does not contain CPS. The OPS has been validated several times in various palliative care.^4–8^

In 2023, modified versions of the OPS (mOPSs) were developed and validated to improve feasibility in patients with advanced cancer in Japan and Korea using the East Asian cross-cultural Collaborative Study to Elucidate the Dying process (EASED).^9^ The mOPS aimed to predict 2-week survival, whereas the original OPS was used to predict 3-week survival. The mOPS has two versions: mOPS-(A) with fewer laboratory tests than OPS and mOPS and (B) without laboratory tests. The mOPS-(A) consists of two symptoms, two signs, and three laboratory tests, with a range of 0–6.5. The mOPS-(B) comprises three symptoms and two signs (range 0–4.0). As reported in a previous study, the original OPS was not available in Taiwan because of missing data on bloodwork.^10^ To date, no studies have examined the applicability of mOPS in countries other than Japan and Korea.

We hypothesized that the mOPS-B could be applied to Taiwanese patients. Because the mOPS-B does not require any blood tests, it would be feasible in patients with shorter survival. Moreover, the mOPS-B could be more accurate than the Karnofsky Performance Status (KPS), which is the cornerstone of prognostication in day-to-day oncology practice.^11^ Thus, this study aimed to validate the mOPS and compare its accuracy with that of the KPS for patients with advanced cancer in Taiwan.

## Methods

### Participants

This was a secondary analysis of an international multicenter prospective observational study called the EASED. This study investigated the dying process and end-of-life care of inpatients with advanced cancer admitted to palliative care units (PCUs). This study was conducted between January 2017 and September 2018. Participants who were newly admitted to participating PCUs during the study period were consecutively enrolled. All observations were performed during routine clinical practice. The inclusion criteria were as follows: age ≥20 years and diagnosis of locally extensive or metastatic cancer. The exclusion criteria were scheduled discharge within 1 week and refusal to participate in the study by the patient or their family.

### Data collection

The physicians prospectively recorded all variables on the first day of admission using structured data collection sheets. We followed up the enrolled patients who were discharged until 6 months after enrollment. Thus, survival time was calculated by subtracting the admission date from the death date. Mortality was defined as all deaths within and outside the PCUs. We treated patients alive at the last follow-up as censored data. Baseline patient demographics included age, sex, primary cancer site, KPS score, Eastern Cooperative Oncology Group (ECOG) performance status, oral intake, dyspnea, leg edema, and drowsiness.

Oral intake was categorized as follows: 0, normal; 1, reduced but more than mouthfuls; and 2, less than mouthfuls. Dyspnea was scored as follows: 0, normal; 1, exertional only; and 2, at rest. As for leg edema, the severity was assessed by measuring the depth upon pressing the skin for a sufficient amount of time in the area between the upper and lower joints and was categorized as follows: 0, none; 1, mild (<5 mm); 2, moderate (5–10 mm); and 3, severe (>10 mm).^12^ In terms of drowsiness, the Integrated Palliative Outcome Scale (IPOS) was used: 0, not at all; 1, slightly; 2, moderately; 3, severely; 4, overwhelmingly; and 5, cannot be assessed because of unconsciousness. We considered a score of 5 as a missing value. The Taiwanese version of the IPOS was validated and reported at an international symposium (Tohoku Forum for Creativity 2020).

The following clinician characteristics were recorded: sex, clinical experience (years), clinical experience in palliative care (years), and number of patients with advanced cancer treated in a year.

### Measurements

The mOPS-A comprises the following seven items: oral intake, dyspnea at rest, ECOG performance status, leukocyte count, serum total bilirubin levels, lactate dehydrogenase levels, and leg edema.^9^ ECOG performance status is a clinician’s rating of physical functional status, similar to KPS. The mOPS-A assigns 1.0 point to oral intake (a few mouthfuls or less), dyspnea at rest, ECOG performance status of 4, leukocytosis (>11,000/μL), elevated serum bilirubin levels (≥2.0 mg/dL), elevated serum lactate dehydrogenase levels (≥502 IU/L), and 0.5 point to leg edema. Thus, a numerical score between 0 and 6.5 was generated. The mOPS-B includes five components: oral intake, dyspnea at rest, ECOG performance status, leg edema, and drowsiness.^9^ However, laboratory data (serum total bilirubin and lactate dehydrogenase levels) needed to calculate mOPS-A scores were lacking in 394 of 407 patients (96.8%). Therefore, we excluded the mOPS-A and validated only the mOPS-B in this study. The mOPS-B assigns 1.0 point to oral intake (a few mouthfuls or less), dyspnea at rest, ECOG performance status of 4, 0.5 point to leg edema, and drowsiness with an IPOS score of ≥1. Thus, a numerical score between 0 and 4 was generated. The patients were divided into two groups according to scores: high group (1.5–4.0), with a predicted survival of <2 weeks and low group (0–1.0), with a predicted survival of ≥2 weeks. The cutoff values were determined to maximize the true-positive rate and minimize the false-negative rate of mOPSs based on a previous study of independent data.^9^

The KPS is a one-item assessment tool for functional impairment.^11^ The level of functional capacity is rated from 0% (dead) to 100% (normal) in 10% increments. The KPS is associated with survival in patients with various types of cancers, although it was not developed as a prognostic tool.^13^ There are no validated prognostic tools to predict 2-week survival other than Prognosis in Palliative care Study predictor model-A (PiPS-A). However, we could not calculate PiPS-A score because we did not assess components of it, such as abbreviated mental test score, heart rate, difficulty swallowing, loss of weight, and global health status. For Palliative Prognostic Score and Palliative Prognostic Index, the time frame differs from 2 weeks. Therefore, KPS was selected for comparison by availability.

### Statistical analysis

First, we performed a descriptive analysis to summarize the baseline patient characteristics.

Second, we calculated the median overall survival and 95% confidence intervals (CI) in each group (high and low groups) and constructed the Kaplan–Meier survival curves for the risk groups classified by each prognostic score.

Third, to assess the discrimination ability of the mOPS-B, we used the area under the receiver operating characteristic curve (AUROC), an approach similarly used in previous studies.^14^ Cutoff values of 1.5 for mOPS-B were based on the previous study.^9^ Meanwhile, a cutoff value of 30 for KPS reflected our data distribution; authors selected the value for matching 2-week survival. Using the cutoff values, we compared the accuracy of 2-week survival prediction by the mOPS-B and KPS. The AUROC is the probability of classifying binary outcomes as its threshold varies and ranges from 0.5 (no discriminatory ability) to 1 (perfect discriminatory ability). We used the mOPS-B scores as continuous variables to calculate the AUROCs.

Fourth, we used a calibration plot (actual vs. predicted) using a logistic regression model.^15^ The scores of both KPS and mOPS-B were considered continuous variables in the calibration assessment.

Fifth, we calculated the additive and absolute net reclassification indices (NRIs) by replacing the KPS with the mOPS-B for 2-week survival. The additive NRI was calculated by summing the percentage of patients experiencing an event (death) and who were correctly reclassified, and the percentage of patients who did not experience an event and who were correctly reclassified. A positive value of the additive NRI indicates better reclassification of patients experiencing an event using newer prediction models, whereas a negative value indicates worse reclassification. The absolute NRI can range from −100% to 100%, representing the proportion of patients who are incorrectly or correctly reclassified. A positive absolute NRI value indicates that the new prediction model works better than the old model, whereas a negative value indicates that the old model works better.

All analyses were performed using the JMP version 17.0 for Windows (SAS, Cary, NC, USA). Statistical significance was set at *p* < 0.05.

### Ethics

All procedures for the primary EASED study were approved by the local institutional review boards (IRBs) of all participating institutions in Taiwan. The study was conducted in accordance with the ethical standards of the Declaration of Helsinki (revised 2013). Informed consent was obtained from the patients or their families (in cases where the patient was incapable of providing consent). The IRBs of the representative institutes in the three sectors approved this secondary analysis: National Taiwan University in Taiwan (201611032RIND).

## Results

### Patient characteristics

**FIG. 1.**
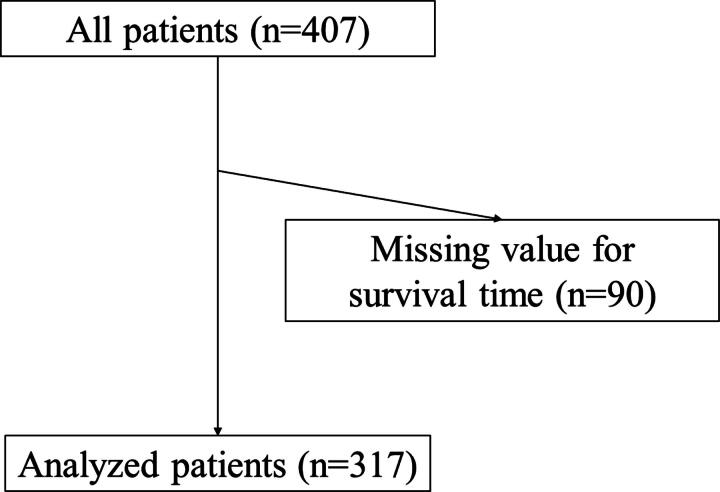
Participant flowchart.

Between January 2017 and September 2018, 407 patients with cancer were recruited from four PCUs in Taiwan. However, 90 patients were excluded because of missing values for survival time. Finally, 317 patients were evaluated (Fig. 1). The baseline patient characteristics are summarized in Table 1. The study included 171 men (54%) and 146 women (46%) with a mean age of 66.3 ± 13.8 (standard deviation, SD) years. The most prevalent primary sites of cancer were the hepatobiliary/pancreas (24%), lungs (18%), and colon/rectum (15%). The median overall survival was 14 days (95% CI: 2–79 days).

**Table 1.** Baseline Characteristics of the Participants (*n* = 317)

Characteristics	*n* (%)
Age (years, mean ± SD)^a^	66.3 ± 13.8
Sex	
Male	171 (53.9)
Female	146 (46.1)
Primary cancer site	
Hepatobiliary/Pancreas	76 (24.0)
Lung	58 (18.3)
Colon/Rectum	48 (15.1)
Head/Neck	35 (11.0)
Esophagus/Stomach	22 (6.9)
Urological system	21 (6.6)
Breast	16 (5.0)
Gynecological system	14 (4.4)
Others	27 (8.5)
ECOG performance status	
1	7 (2.2)
2	20 (6.3)
3	110 (34.7)
4	180 (56.8)
Oral intake	
Normal	77 (24.3)
Reduced but greater than mouthfuls	136 (42.9)
Less than or equal to mouthfuls	104 (32.8)
Dyspnea	
Absent	138 (43.5)
Exertional only	90 (28.4)
At rest	89 (28.1)
Leg edema (edema of lower extremities)	
Absent	159 (50.2)
Mild	77 (24.3)
Moderate	46 (14.5)
Severe	35 (11.0)
Drowsiness^b^	
Absent (Not at all)	78 (24.6)
Slightly	80 (25.2)
Moderately	57 (18.0)
Severely	63 (19.9)
Overwhelmingly	39 (12.3)
Karnofsky Performance Status	
80	4 (1.3)
70	2 (0.6)
60	15 (4.7)
50	23 (7.3)
40	50 (15.8)
30	109 (34.4)
20	53 (16.7)
10	61 (19.2)
Median survival time (days, 95% CI)	14 (2–79)

^a^ Missing value (*n* = 1).

^b^ Drowsiness was assessed by Integrated Palliative Outcome Scale.

CI, confidence interval; ECOG, Eastern Cooperative Oncology Group; SD, standard deviation.

### Clinician characteristics

Table 2 shows the characteristics of the 64 clinicians (32 men and 32 women) who participated in this study. The mean (±SD) durations of the clinical and palliative care careers of Taiwanese clinicians were 5.8 ± 3.5 years and 2.8 ± 3.1 years, respectively.

**Table 2.** General Characteristics of the Participating Clinicians (*n* = 64)

Characteristics	
Sex (male)	32 (50.0)
Career (years; mean ± SD)	5.8 ± 3.5
Career in palliative care (years; mean ± SD)	2.8 ± 3.1
Number of patients with far advanced cancer seen per year (mean ± SD)	111.1 ± 141.3

Data are expressed as number (%) or mean ± SD.

### Median survival time of each risk group according to prognostic score

**FIG. 2.**
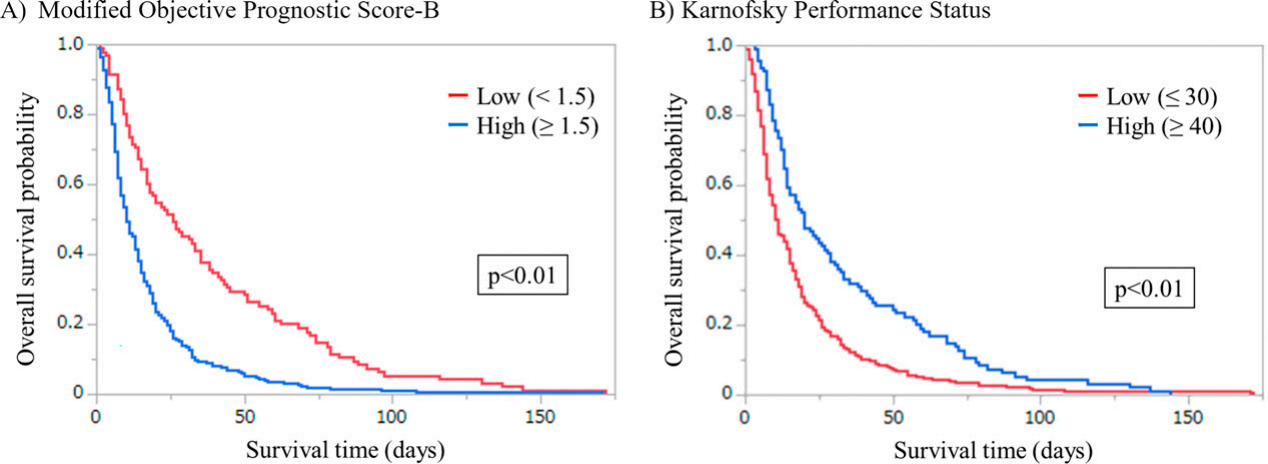
Kaplan–Meier survival curves for overall survival according to modified Objective Prognostic Score-B and Karnofsky Performance Status from the time of enrollment. **(A)** Modified Objective Prognostic Score-B. **(B)** Karnofsky Performance Status. *p*-Values were derived using a log-rank test.

The median survival times observed according to mOPS-B scores were 26 days (95% CI: 17–35 days) in the low group (0–1) and 10 days (95% CI: 8–13 days) in the high group (1.5–4.0). The median survival times observed according to KPS scores were 20 days (95% CI: 14–29 days) in the high (KPS: 40–80) and 11 days (95% CI: 9–14 days) in the low (KPS: 10–30) group. Figure 2 shows the survival curves from the time of enrollment for each prognostic score. Prominent discrimination in the Kaplan–Meier plots according to cutoff values was observed in the mOPS-B (*p* < 0.01) and KPS (*p* < 0.01).

### Discrimination and calibration

**FIG. 3.**
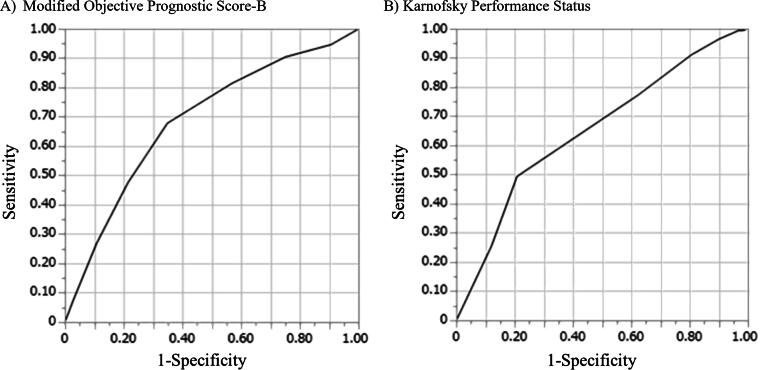
Receiver operating characteristic curve for the modified Objective Prognostic Score-B and Karnofsky Performance Status to predict 2-week survival. **(A)** Modified Objective Prognostic Score-B. **(B)** Karnofsky Performance Status.

Table 3 summarizes the discrimination ability of the mOPS-B and KPS for predicting two-week survival. The AUROC of mOPS-B was 0.69 (95% CI, 0.63 − 0.74) with high sensitivity (0.82), and that of KPS was 0.65 (95% CI, 0.59 − 0.71) with good sensitivity (0.77). Thus, mOPS-B and KPS demonstrated similar discriminatory abilities with no significant difference. Figure 3 shows the ROC curves for the mOPS-B and KPS scores.

**FIG. 4.**
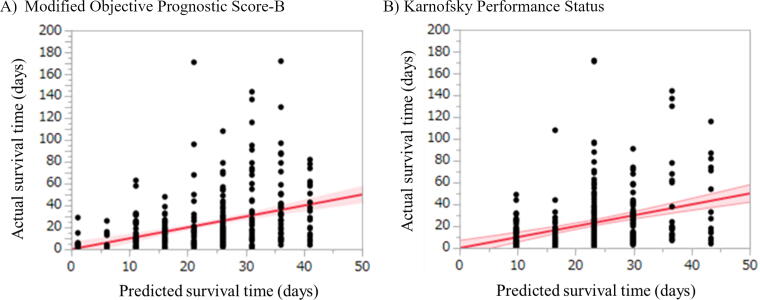
Calibration plots of the modified Objective Prognostic Score-B and Karnofsky Performance Status. **(A)** Modified Objective Prognostic Score-B. **(B)** Karnofsky Performance Status. The decile on the *x*-axis represents the survival time predicted by the modified Objective Prognostic Score-B or Karnofsky Performance Status, and the decile on the *y*-axis represents the actual survival time. The reference line (red line) indicates a perfect model in which the actual survival time is equal to the predicted survival time.

The calibration plot is presented in Figure 4, in which perfect calibration is represented by the reference line in red. Grossly, mOPS-B seemed to have a tighter band around the reference line than KPS. However, both mOPS-B and KPS did not show satisfactory agreement between the actual and predicted survival times.

Table 4 shows that when we replaced KPS with mOPS-B, the additive NRI value was 39%, and the absolute NRI was 22% for 2-week survival. A positive additive NRI indicates a better reclassification ratio, whereas a positive absolute NRI value indicates an overall better reclassification, which is adjusted for the total number of patients. The results indicated that the mOPS-B better predicted 2-week survival than the KPS.

**Table 3.** Performance and Discrimination Values of the Modified Objective Prognostic Score-B and Karnofsky Performance Status to Predict 2-Week Survival

Prognostic index	Prevalence *n* (%)	Sensitivity	Specificity	PPV	NPV	OA	AUROC (95% CI)
mOPS-B (≥1.5)	168 (53.0)	0.82	0.43	0.62	0.67	0.63	0.69 (0.63–0.74)
KPS (≤30)	168 (53.0)	0.77	0.38	0.58	0.60	0.59	0.65 (0.59–0.71)

AUROC, area under the receiver operating characteristic curve; KPS, Karnofsky Performance Status; mOPS, modified Objective Prognostic score; NPV, negative predictive value; OA, overall accuracy; PPV, positive predictive value.

**Table 4.** Net Reclassification Indices of the Modified Objective Prognostic Score-B and Karnofsky Performance Status

	Patients with an event (*n*)	Patients without an event (*n*)	Additive NRI	Absolute NRI (%)
Replacement of KPS with mOPS-B for 2-week survival				
	Total	168	149	39.47	22.08
	Correct reclassification	114	41		
	Incorrect reclassification	15	70		
	Net reclassification	99	−29		

Additive NRI: (net reclassification/total number of patients with an event) × 100 + (net reclassification/total number of patients without an event) × 100.

Absolute NRI: (net reclassification of patients with an event + net reclassification of patients without an event)/total number of patients × 100.

NRI, net reclassification index.

## Discussion

The mOPS-B demonstrated good ability to distinguish between groups based on 2-week survival among inpatients with advanced cancer in Taiwan. In addition, the accuracy of mOPS-B was comparable to that of KPS in this study. The mOPS-B better predicted 2-week survival than KPS., although neither achieved satisfactory results in the calibration plots.

In a previous study, mOPS-B showed an acceptable AUROC (0.74–0.75).^9^ The AUROC reflects how able the mOPS-B is to differentiate between better and poorer survival groups. Our results revealed slightly lower AUROC values (0.69) than that in a previous study. This observation has a few possible explanations. First, our patients had a shorter median survival time (14 days) than those reported in a previous study (19 days). We assume that the difference in survival time may have caused the lower accuracy here. Second, the previous validation study included a larger population (*n* = 1,796) than the current study (*n* = 371).

By contrast, the mOPS-B demonstrated higher sensitivity (0.82) than that of the previous study (0.72–0.73). Thus, if the mOPS-B score is <1.5, the probability of survival within 2 weeks is likely high: 67.4% (64/95) of patients with the mOPS-B score of <1.5 could survive more than 2 weeks in current study. Also the mOPS-B demonstrated higher sensitivity than KPS for predicting 2-week survival in this study. Unfortunately, the calibration plots of the mOPS-B presented a lower location than those reported in a previous study. It implies that actual accuracy may vary according to individual cases. Therefore, Taiwanese clinicians should be aware of the limitations of the mOPS-B in predicting 2-week survival.

Surprisingly, both mOPS-B and KPS demonstrated a similar differentiating ability for groups with better and poorer survival. The mOPS-B has five variables, whereas KPS is a performance status item. Initially, we assumed that the mOPS-B was more accurate than the KPS. The mOPS-B includes ECOG performance status, which is equivalent to the KPS as a performance scale. Other items of the mOPS-B (oral intake, dyspnea at rest, drowsiness, and leg edema) may reflect functional impairment that influences the performance status in the KPS. Therefore, the results may be similar. In terms of clinical implications, our results showed positive NRI values, suggesting that mOPS-B predicted 2-week survival more accurately than KPS.

As expected, the mOPS-A was not feasible for Taiwanese patients. In Taiwan, patients in the PCUs were admitted when they were close to death.^16^ Therefore, Taiwanese physicians providing palliative care tend to not order blood tests in PCUs, although laboratory data before admission are available.^17^ To calculate mOPS-A, the results of liver function tests (lactate dehydrogenase and bilirubin) and complete blood counts (white blood cells and lymphocytes) are needed. In Taiwan, complete blood counts might be obtained at outpatient clinics because they are basic laboratory tests for patients with cancer, whereas liver and renal function tests might not be performed. Hence, the feasibility of calculating the mOPS-A was very low.

Our study has several limitations. First, it was conducted in PCUs in Taiwan. Therefore, our findings may not be generalizable to different palliative care settings, such as general wards, home hospice care, or other countries. Second, the KPS, clinicians’ prediction of survival, and mOPS (symptoms, signs, ECOG PS) are all rated by physicians. Therefore, they may be equally susceptible to assessment bias and random error. Third, the median survival time of the enrolled patients was shorter (14 days) than that reported in a previous study (19 days). The mOPS-B needs to be further evaluated in patients with advanced cancer with various survival times. Fourth, the mOPS-A was not available for Taiwanese patients. Based on our results, application of mOPS in various settings is warranted.

In conclusion, here we assessed the validity of the mOPS-B for predicting death within 2 weeks among Taiwanese patients. The mOPS-B demonstrated good differentiating ability for predicting 2-week survival in patients with advanced cancer in PCUs. Therefore, the mOPS-B may serve better than the KPS as a screening tool for admission to PCUs in Taiwan because it was more accurate at predicting 2-week survival.

## Data Availability

The data supporting the findings of this study are available from the corresponding author, S-Y.S., upon reasonable request. All authors agree to provide data to the journal for review, if needed.

## Abbreviations Used

AUROC: area under the receiver operating characteristic curve
CI: confidence intervals
CPS: clinicians' prediction of survival
EASED: East Asian cross-cultural collaborative Study to Elucidate the Dying process
ECOG: Eastern Cooperative Oncology Group
ICMJE: International Committee of Medical Journal Editors
IPOS: Integrated Palliative Outcome Scale
IRB: institutional review board
KPS: Karnofsky Performance Status
mOPS: modified versions of the Objective Prognostic Score
NRI: net reclassification indice
OPS: Objective Prognostic Score
PaP: Palliative Prognostic Score
PCU: palliative care unit
PiPS: Prognosis in Palliative care Study predictor model
PPI: Palliative Prognostic Index
SD: standard deviation
